# Ptbp2 re-expression rescues axon growth defects in Smn-deficient motoneurons

**DOI:** 10.3389/fnmol.2024.1393779

**Published:** 2024-08-23

**Authors:** Saeede Salehi, Abdolhossein Zare, Gayatri Gandhi, Michael Sendtner, Michael Briese

**Affiliations:** Institute of Clinical Neurobiology, University Hospital Würzburg, Würzburg, Germany

**Keywords:** spinal muscular atrophy, SMN, axonal RNA transport, axonal translation, axon growth, Ptbp2

## Abstract

Spinal muscular atrophy (SMA) is a neuromuscular disorder caused by mutations or deletions in the survival motoneuron 1 (*SMN1*) gene, resulting in deficiency of the SMN protein that is essential for motoneuron function. Smn depletion in mice disturbs axonal RNA transport and translation, thereby contributing to axon growth impairment, muscle denervation, and motoneuron degeneration. However, the mechanisms whereby Smn loss causes axonal defects remain unclear. RNA localization and translation in axons are controlled by RNA-binding proteins (RBP) and we recently observed that the neuronal RBP Ptbp2 modulates axon growth in motoneurons. Here, we identify Smn as an interactor of Ptbp2 in the cytosolic compartments of motoneurons. We show that the expression level of Ptbp2 is reduced in axons but not in the somata of Smn-depleted motoneurons. This is accompanied by reduced synthesis of the RBP hnRNP R in axons. Re-expression of Ptbp2 in axons compensates for the deficiency of Smn and rescues the defects in axon elongation and growth cone maturation observed in Smn-deficient motoneurons. Our data suggest that Ptbp2 and Smn are components of cytosolic mRNP particles, contributing to the precise spatial and temporal control of protein synthesis within axons and axon terminals.

## Introduction

Spinal muscular atrophy (SMA) is a severe neuromuscular disorder characterized by lower motoneuron degeneration and caused by reduced expression of the survival motor neuron (SMN) protein due to mutations or deletions in the *SMN1* gene (Lefebvre et al., 1995). In the cytosol, SMN assembles spliceosomal small nuclear ribonucleoproteins (snRNPs) (Fischer et al., 1997; Liu et al., 1997; Pellizzoni et al., 1998). Additionally, granules containing Smn have been observed in axons and axon terminals of motoneurons (Jablonka et al., 2001; Giavazzi et al., 2006; Dombert et al., 2014). Several RNA binding proteins (RBPs) interact with SMN including hnRNP R, and this interaction is necessary for the transport of mRNAs such as *Actb* mRNA encoding β-actin into axons (Rossoll et al., 2003; Glinka et al., 2010). Impaired axonal RNA localization and translation have been linked to SMA, and motoneurons cultured from an SMA mouse model show defects in axon growth (Rossoll et al., 2003; Jablonka et al., 2007). However, the molecular mechanism underlying the axon growth defects caused by Smn deficiency remains unclear.

Recently, we demonstrated that Ptbp2, a neuronal RBP, facilitates the axonal localization and translation of the *Hnrnpr* transcript encoding hnRNP R in motoneurons, thereby supporting axon growth (Salehi et al., 2023). Here, we show that Smn is associated with Ptbp2 not only in the cell body but also in axons and growth cones of motoneurons, and this interaction is RNA-independent. We found that the level of Ptbp2 protein is significantly reduced in axons but not cell bodies of Smn knockout motoneurons cultured from an SMA mouse model. The reduction in Ptbp2 was accompanied by decreased levels of hnRNP R in axonal compartments of Smn-deficient motoneurons. Re-introducing Ptbp2 could rescue axon elongation and growth cone maturation defects in Smn-depleted motoneurons. Altogether, our data suggest that Smn and Ptbp2 are components of cytosolic granules in motoneurons that control axonal localization and translation of proteins such as hnRNP R.

## Materials and methods

### Animals and ethical approval

All of the experimental procedures in this study were performed according to the regulations on animal protection of the German federal law and the Association for Assessment and Accreditation of Laboratory Animal Care, in agreement with and under the control of the local veterinary authority. Mice were housed in the animal facility of the Institute of Clinical Neurobiology at the University Hospital of Wuerzburg. The CD1 and *Smn* knockout mice were maintained on a 12 h/12 h day/night cycle under controlled conditions at 20–22°C and 55–65% humidity with food and water in abundant supply.

### Isolation and enrichment of primary embryonic mouse motoneurons

Isolation and enrichment of primary mouse motoneurons were performed as previously described (Wiese et al., 2010). Lumbar spinal cords were dissected from E13 mouse embryos, and motoneurons were isolated by panning using a p75^NTR^ antibody. Cells were plated on coverslips or culture dishes coated with poly-DL-ornithine hydrobromide (PORN) (P8638, Sigma) and laminin-111 (23017-015, Thermo Fisher Scientific). Motoneurons were maintained at 37°C, 5% CO_2_ in neurobasal medium (Gibco) supplemented with 2% B27 (Gibco), 2% heat-inactivated horse serum (Linaris), 500 μM GlutaMAX (Gibco) and 5 ng/ml of brain-derived neurotrophic factor (BDNF). Medium was replaced one day after plating and then every other day.

### Plasmid construction

To generate the construct for expressing EGFP-tagged Ptbp2 (EGFP-Ptbp2), the mouse Ptbp2 coding sequence and the coding sequence of EGFP were PCR-amplified from mouse cDNA and the pSIH-HI plasmid, respectively. Subsequently, the PCR products were inserted into pSIH-H1 digested with SalI (FD0644, Thermo Fisher Scientific) and NheI (FD0973, Thermo Fisher Scientific) using the NEBuilder HiFi DNA Assembly Cloning Kit (New England Biolabs).

### Lentiviral transduction

Lentiviral particles were packaged in HEK293TN cells (System Biosciences, cat. no. LV900A-1) cells with pCMV-pRRE, pCMV-pRSV, and pCMV-pMD2G as described before (Subramanian et al., 2012). Transduction was performed by incubation of motoneurons with lentiviruses in a total volume of 50 μl for 10 min at room temperature before plating at on day *in vitro* (DIV) 0.

### Co-immunoprecipitation

Primary mouse motoneurons were grown on laminin-111-coated 6 cm dishes for 7 DIV. Cells were washed once with Dulbecco’s Phosphate Buffered Saline (DPBS, without MgCl_2_, CaCl_2_; D8537, Sigma-Aldrich) and lysed in lysis buffer (10 mM HEPES pH 7.0, 100 mM KCl, 5 mM MgCl_2_, 0.5% NP-40) on ice for 15 min and cleared via centrifugation at 20,000 × *g* for 15 min at 4°C. The supernatant was then divided into two microtubes and 0.1 μg RNase A (EN0531, Thermo Fisher Scientific) was added to the microtube labeled +RNase and incubated for 15 min at room temperature. Protein G Dynabeads were bound to either 1 μg of normal rabbit IgG (500-P00, PeproTech) or 1 μg of anti-Ptbp2 antibody (55186-1-AP, Proteintech) by rotating for 60 min at room temperature. 300 μl lysate was added to the antibody-bound beads and rotated for overnight at 4°C. Beads were washed twice with 500 μl lysis buffer and proteins were eluted in 1 × Laemmli buffer. Proteins were size-separated by SDS-PAGE and analyzed by immunoblotting.

### Proximity ligation assay (PLA)

PLA was carried out using the Duolink In Situ Orange Starter Kit Mouse/Rabbit (DUO92102, Sigma-Aldrich) according to the manufacturer’s recommendations. Briefly, motoneurons were grown for 6 DIV on laminin-111-coated glass coverslips and washed twice with DPBS. Cells were fixed in paraformaldehyde lysine phosphate (PLP) buffer (pH 7.4) containing 4% paraformaldehyde (PFA) (28908, Thermo Fisher Scientific), 5.4% glucose and 0.01 M sodium metaperiodate for 10 min, then permeabilized. After permeabilization and washing, cells were blocked in blocking buffer for 1 h at 37°C and incubated with antibodies against Ptbp2 (1:100; 55186-1-AP, Proteintech) and Smn (1:100; 610647, BD Biosciences) diluted in blocking buffer overnight at 4°C. PLA probes were applied at 1:5 dilution for 1 h at 37°C, followed by ligation and amplification for 30 and 100 min, respectively. Cells were fixed again for 10 min at room temperature in PLP, washed with DPBS, and stained with FITC-conjugated anti-Tubb3 antibody (130-131-158, Miltenyi Biotec).

### Puromycylation-PLA

Motoneurons isolated from Smn^–/–^,*SMN2*^tg/tg^ and +/+ mice were grown for 6 DIV on laminin-111-coated glass coverslips. Cells were treated with 10 μg/ml puromycin (Sigma-Aldrich, P8833) supplemented in the medium for 8 min at 37°C in a cell culture incubator. In negative control experiments, puromycin was omitted. Cells were washed twice with prewarmed Hanks’ Balanced Salt Solution (HBSS; Gibco) and fixed for 10 min in PLP. After fixation, cells were washed and permeabilized for a proximity ligation assay (PLA) using antibodies against puromycin (Sigma-Aldrich, MABE343, 1:200 dilution) and the N-terminus of hnRNP R (Sigma-Aldrich, HPA026092, 1:200 dilution).

### Immunofluorescence staining

Motoneurons were cultured on laminin-111- and PORN-coated glass coverslips for 7 DIV. Cells were washed twice with DPBS and fixed with 4% paraformaldehyde (PFA) at room temperature for 15 min followed by permeabilization with 0.3% Triton X-100 at room temperature for 20 min. Cells were washed three times with DPBS, blocked in a blocking buffer containing 4% BSA at room temperature for 1 h and then incubated in primary antibodies [anti-Ptbp2, 1:250 (55186-1-AP, Proteintech); anti-tubulin, 1:500 (T5168, Sigma-Aldrich); anti-tau, 1:500 (T6402, Sigma-Aldrich)] at 4°C overnight. This was followed by incubation with secondary antibodies [all at 1:500; for anti-tubulin: donkey polyclonal anti-mouse (DyLight 488-conjugated; SA5-10166, Thermo Fisher Scientific); for anti-Ptbp2 and anti-tau: donkey polyclonal anti-rabbit (Alexa Fluor^®^ 647-conjugated; A31573, Thermo Fisher Scientific)] at room temperature for 1 h and counterstaining with 4′,6-diamidino-2-phenylindole (DAPI). Alexa Fluor 546 phalloidin (A22283, Invitrogen) was added at 1:50 in DPBS during incubation with secondary antibodies. Coverslips were washed and mounted using FluorSave Reagent (Merck, 345789) and subsequently imaged.

### Image acquisition and data analysis

Images were acquired on an Olympus Fluoview 1000 confocal system equipped with the following objectives: 10 × (NA: 0.25), 20 × (NA: 0.75), 40 × (oil differential interference contrast, NA: 1.30), or 60 × (oil differential interference contrast, NA: 1.35). Fluorescence excitation was achieved with using 405, 473, 559, and 633 nm lasers. Images were obtained with the corresponding Olympus FV10-ASW (RRID:SCR_014215) imaging software for visualization. The resulting images (Olympus.oib format) were processed by maximum intensity projection and were adjusted in brightness and contrast using ImageJ as part of the Fiji package (Schindelin et al., 2012).

For quantification of immunofluorescence signals of Ptbp2, raw images were projected using ImageJ and mean gray values were measured after background subtraction. For axon length measurements, transduced motoneurons were plated on laminin-111 and immunostained at DIV 7 with an anti-tau antibody. The images were acquired with a Keyence BZ-8000K fluorescence microscope equipped with a standard color camera using a 20 × 0.7-NA objective. The length of the longest axon branch was quantified using ImageJ software. Axon collaterals were not considered for the analysis. Motoneurons were only scored when designated axons were at least three times longer than the corresponding dendrites ensuring an unambiguous distinction between axons and dendrites. For growth cone size analysis, cells were plated on laminin-221 (CC085; Merck) for 7 DIV and stained with anti-tau and phalloidin. The area of the growth cone was measured using ImageJ software. Images from control and *Smn* knockout motoneurons were acquired with identical settings (laser intensity and photomultiplier voltage).

### Protein extraction and western blotting

Total protein was extracted from primary motoneurons with RIPA buffer (50 mM Tris-HCl pH 7.4, 150 mM NaCl, 1% NP-40, 0.05% sodium deoxycholate, 0.1% SDS). Protein concentration was quantified using a BCA protein assay kit (23227, Thermo Fisher Scientific). Equal amounts of proteins were size-separated by SDS-PAGE gel electrophoresis followed by transfer onto nitrocellulose membrane and immunoblotting with primary antibodies [anti-Ptbp2, 1:2,000; anti-Smn, 1:2,000; anti-Histone H3, 1:10,000 (ab1791, Abcam); anti-β-actin, 1:10,000 (GTX26276, GeneTex)] diluted in Tris-buffered saline with Tween 20 (TBST) (50 mM Tris–HCl pH 7.6, 150 mM NaCl, 1% Tween 20) overnight at 4°C. Following three washes with TBST, peroxidase-conjugated secondary antibodies [all at 1:10,000; for anti-Ptbp2 and anti-Histone H3: mouse monoclonal anti-rabbit (211-032-171, Jackson ImmunoResearch); for anti-β-actin and anti-Smn: goat polyclonal anti-mouse IgG (115-035-174, Jackson ImmunoResearch)] were added for 1 h at room temperature. Blots were washed three times with TBST and incubated with ECL Western blotting substrate (32106, Thermo Fisher Scientific) followed by exposure on X-ray film (Fuji super RX). Blots were scanned and quantified by densitometry analysis using ImageJ.

### Statistics and reproducibility

All statistical analyses were performed using GraphPad Prism version 9 for Windows (GraphPad Software, San Diego, CA, USA). No statistical method was used to predetermine the sample size. No data were excluded from the analyses. Two groups were compared using unpaired two-tailed Student’s *t*-test, two-tailed one-sample *t*-test or Mann–Whitney test. For multiple independent groups, Kruskal-Wallis test with Dunn’s multiple comparisons test. Details of replicate numbers, quantification, and statistics for each experiment are specified in the figure legends.

## Results

### Smn is associated with Ptbp2 in axons of motoneurons

**FIGURE 1:**
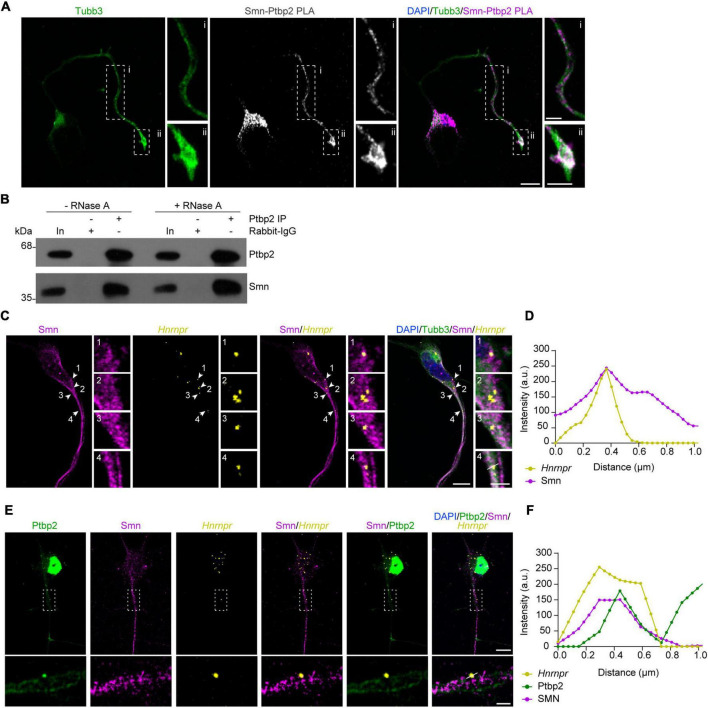
Ptbp2 interacts with Smn in motoneurons. **(A)** Representative images of Smn-Ptbp2 PLA signal in cultured motoneurons at DIV 6 using anti-Smn and anti-Ptbp2 antibodies. Motoneuron morphology was visualized with anti-Tubb3 antibody. Scale bars, 10 and 5 μm (magnified areas). **(B)** Co-immunoprecipitation of Smn by anti-Ptbp2 from motoneuron lysate pre-treated with RNase A as indicated. **(C)** Representative images showing Smn immunofluorescence and *Hnrnpr* FISH in cultured motoneurons at DIV 6. Arrowheads indicate colocalization of Smn and *Hnrnpr* in granules. Scale bars, 10 and 2 μm (magnified areas). **(D)** Fluorescence intensity profiles of Smn and *Hnrnpr* at the location indicated by arrow 4 in **(C)**. **(E)** Representative images showing Ptbp2 and Smn immunofluorescence and *Hnrnpr* FISH in cultured motoneurons at DIV 6. Scale bars, 10 and 2 μm (magnified areas). **(F)** Fluorescence intensity profiles of Ptbp2, Smn and *Hnrnpr* at the location indicated by a line in **(E)**.

Guided by our recent study demonstrating that depletion of Ptbp2 leads to axon growth defects similar to Smn-deficient motoneurons (Salehi et al., 2023), we investigated whether Ptbp2 is associated with Smn in cultured primary mouse motoneurons. For this purpose, we evaluated the interaction between Ptbp2 and Smn *in situ* by performing a proximity ligation assay (PLA) using antibodies against Ptbp2 and Smn. We observed that the Ptbp2-Smn PLA signal was detectable in the cytosol of the somata as well as in axons and growth cones of cultured motoneurons (Figure 1A). As negative controls, no signal was detected when either anti-Ptbp2 or anti-Smn antibody was omitted (Supplementary Figure 1). To further validate the association between Ptbp2 and Smn, we performed immunoprecipitation from motoneuron lysates using anti-Ptbp2 antibody and evaluated Smn co-immunoprecipitation by immunoblot analysis. We assessed the RNA-dependence of the interaction by treating the motoneuron lysate with RNase A. We found that Smn co-precipitated with Ptbp2 without and with RNase A treatment, indicating that the interaction between Ptbp2 and Smn is RNA-independent (Figure 1B). Together, these data show that Smn interacts with Ptbp2 in axons of motoneurons.

We previously showed that Ptbp2 and *Hnrnpr* mRNA are components of cytosolic mRNP particles in axons of motoneurons. To investigate whether Smn is associated with this complex, we visualized *Hnrnpr* mRNA by fluorescent *in situ* hybridization (FISH) and Smn by immunostaining in motoneurons. We observed that Smn-positive punctae were in close proximity to the FISH signal for *Hnrnpr* mRNA in axons (Figures 1C, D). Next, we assessed whether Smn is associated with Ptbp2 complexes containing *Hnrnpr* mRNA in the axons of motoneurons. To do so, we performed FISH for *Hnrnpr* visualization together with Smn and Ptbp2 immunostaining. Smn-positive punctae were observed close to the Ptbp2-positive punctae that co-localized with *Hnrnpr* mRNA in axons (Figures 1E, F). These data suggest that Smn is associated with cytosolic Ptbp2 complexes containing *Hnrnpr* mRNA.

### Ptbp2 is reduced in axons of Smn-deficient motoneurons

**FIGURE 2:**
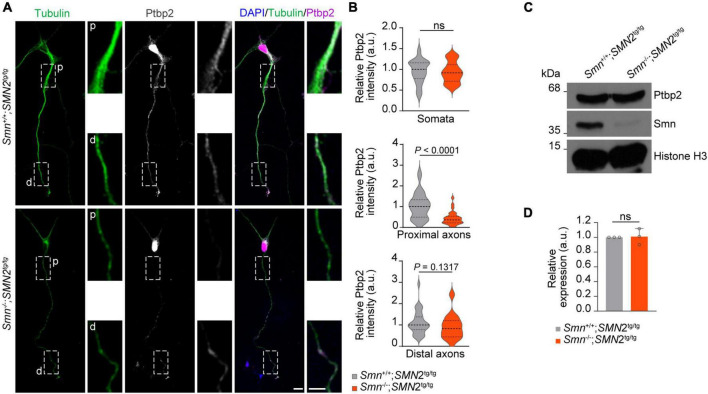
Reduction of Ptbp2 in axons of Smn-deficient motoneurons. **(A)** Immunofluorescence imaging of Ptbp2 in motoneurons cultured from *Smn*^+/+^; *SMN2*^tg/tg^ and *Smn*^–/–^; *SMN2*^tg/tg^ mice at DIV 7. Scale bars, 10 and 5 μm (magnified areas). **(B)** Ptbp2 immunosignals in somata, proximal (p) and distal (d) axons. *n* (*Smn*^+/+^; *SMN2*^tg/tg^) = 31 and *n* (*Smn*^–/–^; *SMN2*^tg/tg^) = 28 motoneurons from three biological replicates. Mann–Whitney and unpaired two-tailed Student’s *t*-test. **(C)** Immunoblot of Ptbp2 in *Smn*^+/+^; *SMN2*^tg/tg^ and *Smn*^–/–^; *SMN2*^tg/tg^ motoneurons at DIV 7. Histone H3 was used as loading control. **(D)** Quantitative analysis of Western blots as shown in **(C)** for Ptbp2. Two-tailed one-sample *t*-test. Data are mean ± s.d. of *n* = 3 biological replicates.

Having shown that Ptbp2 is associated with Smn in axons of motoneurons, we next addressed the question whether the axonal localization of Ptbp2 is regulated by Smn. To do so, we performed Ptbp2 immunostaining on primary motoneurons cultured from a severe SMA mouse model. In these mice, deletion of murine *Smn* is partially compensated for by expression of human SMN from an *SMN2* transgene (Monani et al., 2000). We observed that the level of Ptbp2 was significantly reduced in proximal axons of motoneurons cultured from *Smn*^–/–^; *SMN2*^tg/tg^ mice while Ptbp2 levels in the somata were unchanged. Distally, we observed a tendency toward Ptbp2 reduction in axons of Smn-deficient motoneurons (Figures 2A, B). In line with this result, the total level of Ptbp2 was not affected in *Smn*-deficient motoneurons (Figures 2C, D). Thus, Smn regulates the axonal localization of Ptbp2.

### Loss of Smn affects the local synthesis of hnRNP R in axons

**FIGURE 3:**
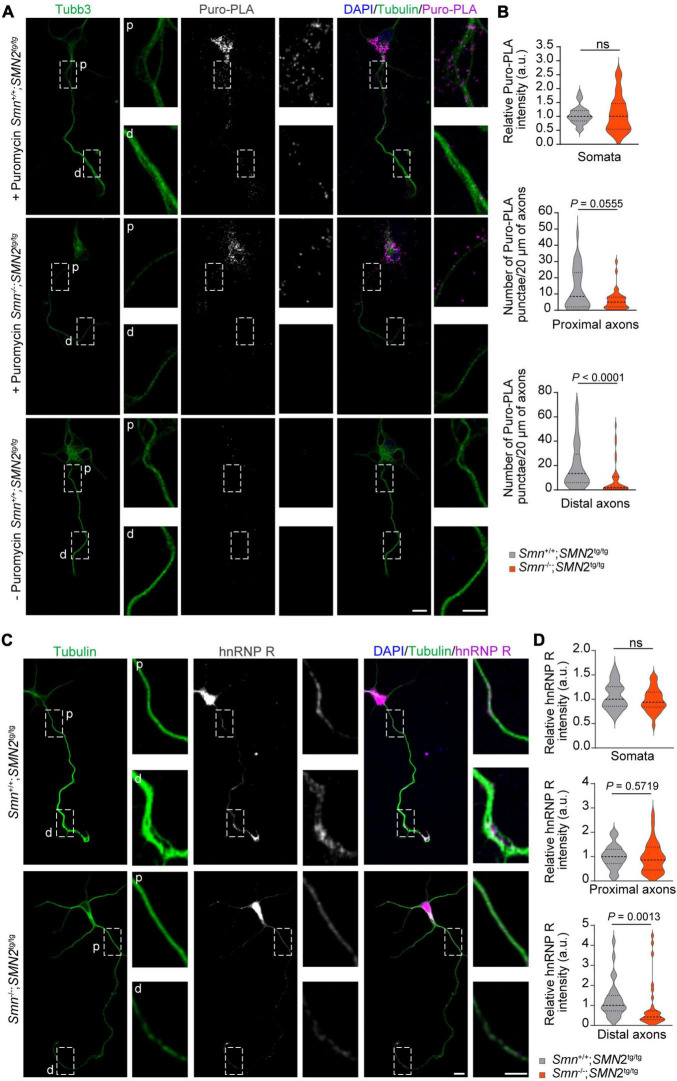
Reduction of hnRNP R in axons of Smn-deficient motoneurons. **(A)** Puro-PLA of hnRNP R in control and Smn-deficient motoneurons. Scale bars, 10 and 5 μm (magnified areas). **(B)** Quantification of relative Puro-PLA intensity in somata and the number of Puro-PLA punctae in 20 μm of proximal and distal axons of motoneurons cultured from *Smn*^+/+^; *SMN2*^tg/tg^ and *Smn*^–/–^; *SMN2*^tg/tg^ mice at DIV 6. *n* (*Smn*^+/+^; *SMN2*^tg/tg^) = 34 and *n* (*Smn*^–/–^; *SMN2*^tg/tg^) = 36 motoneurons from three biological replicates. Mann–Whitney and unpaired *t*-test. **(C)** Immunofluorescence imaging of hnRNP R in motoneurons cultured from *Smn*^+/+^; *SMN2*^tg/tg^ and *Smn*^–/–^; *SMN2*^tg/tg^ mice at DIV 7. Scale bars, 10 and 5 μm (magnified areas). **(D)** hnRNP R immunosignals in somata, proximal and distal axons. *n* (*Smn*^+/+^; *SMN2*^tg/tg^) = 28 and *n* (*Smn*^–/–^; *SMN2*^tg/tg^) = 31 motoneurons from three biological replicates. Mann–Whitney and unpaired *t*-test.

We previously showed that Ptbp2 promotes axonal hnRNP R translation in motoneurons (Salehi et al., 2023). Therefore, we investigated whether the reduction of Ptbp2 in axons of Smn-depleted motoneurons affects the axonal levels of hnRNP R. For this purpose, we first performed puromycin treatment coupled with PLA (Puro-PLA) to measure newly synthesized hnRNP R in both control and Smn-depleted motoneurons. In this assay, puromycin is incorporated into nascent polypeptides such that PLA with antibodies against the N-terminus of hnRNP R and puromycin can visualize newly synthesized hnRNP R (Tom Dieck et al., 2015). We observed that the number of hnRNP R Puro-PLA punctae was significantly reduced in the distal axons of Smn-depleted motoneurons (Figures 3A, B). We also found a tendency for a reduced number of Puro-PLA punctae in proximal axons of Smn-depleted motoneurons but there was no change in somata (Figures 3A, B). These data suggest that Smn is involved in axonal hnRNP R translation. Consistent with these results, hnRNP R immunostaining revealed that the level of hnRNP R was reduced in the distal axons of Smn-deficient motoneurons while it remained unchanged in the somata (Figures 3C, D). Together, our findings suggest that Smn deficiency affects local hnRNP R synthesis in axons.

### Re-expression of Ptbp2 rescues axon growth in Smn-deficient motoneurons

**FIGURE 4:**
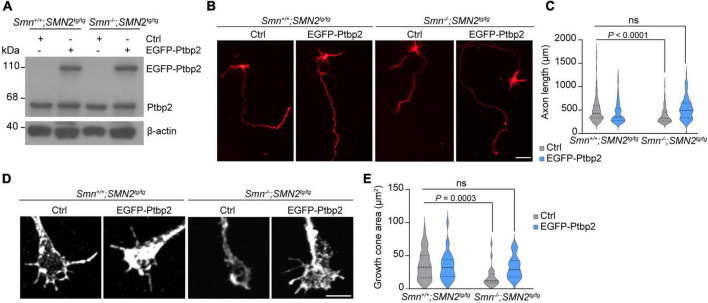
Re-introducing Ptbp2 restores impaired axon growth in *Smn*-deficient motoneurons. **(A)** Immunoblot analysis of Ptbp2 in *Smn*^+/+^; *SMN2*^tg/tg^ and *Smn*^–/–^; *SMN2*^tg/tg^ motoneurons transduced with lentivirus expressing either EGFP (Ctrl) or EGFP-Ptbp2 at DIV 7. β-actin was used as a loading control. **(B)** Cultured DIV 7 motoneurons from *Smn*^+/+^; *SMN2*^tg/tg^ and *Smn*^–/–^; *SMN2*^tg/tg^ mice immunostained for tau. Scale bar, 50 μm. **(C)** Quantification of axon lengths. Kruskal-Wallis test with Dunn’s multiple comparisons test. For Ctrl, *n* (*Smn*^+/+^; *SMN2*^tg/tg^) = 462 and *n* (*Smn*^–/–^; *SMN2*^tg/tg^) = 229 motoneurons; for EGFP-Ptbp2, *n* (*Smn*^+/+^; *SMN2*^tg/tg^) = 398 and *n* (*Smn*^–/–^; *SMN2*^tg/tg^) = 99 from three biological replicates. **(D)** Representative images of growth cones of *Smn*^+/+^; *SMN2*^tg/tg^ and *Smn*^–/–^; *SMN2*^tg/tg^ motoneurons expressing either EGFP (Ctrl) or EGFP-Ptbp2 at DIV 7. **(E)** Quantification of growth cone size. Kruskal-Wallis test with Dunn’s multiple comparisons test. For Ctrl, *n* (*Smn*^+/+^; *SMN2*^tg/tg^) = 44 and *n* (*Smn*^–/–^; *SMN2*^tg/tg^) = 25 motoneurons; for EGFP-Ptbp2, *n* (*Smn*^+/+^; *SMN2*^tg/tg^) = 33 and *n* (*Smn*^–/–^; *SMN2*^tg/tg^) = 25 from three biological replicates.

Previous studies have shown that loss of Smn in SMA mouse models and patients with SMA results in axonal and synaptic defects (Jablonka et al., 2022). Furthermore primary motoneurons cultured from *Smn*^–/–^; *SMN2*^tg/tg^ exhibit decreased growth cone size and impaired axon elongation (Rossoll et al., 2003; Jablonka et al., 2007). To examine whether re-introducing Ptbp2 can rescue impaired axon growth in Smn-deficient motoneurons, primary motoneurons cultured from *Smn*^–/–^; *SMN2*^tg/tg^ and *Smn*^+/+^; *SMN2*^tg/tg^ mice were transduced with lentiviruses expressing an EGFP-Ptbp2 fusion protein (Figure 4A). We found that re-expression of Ptbp2 could restore axon length and growth cone size in Smn-depleted motoneurons (Figures 4B–E). These results indicate that the reduction of axonal Ptbp2 contributes to the impairment of axon growth in Smn-deficient motoneurons.

## Discussion

The SMN protein has a canonical function in spliceosomal snRNP biogenesis in the cytoplasm (Fischer et al., 1997). This, however, raises the question why loss of SMN in SMA particularly affects lower motoneurons in the spinal cord (Briese et al., 2005). In motoneurons, SMN is also present in axons and growth cones (Jablonka et al., 2001; Zhang et al., 2003, 2006; Giavazzi et al., 2006). SMN is associated with a variety of RBPs known to be involved in mRNA transport, stability, and local translation in neurons including hnRNP R, hnRNP Q, FMRP and HuD (Rossoll et al., 2002; Piazzon et al., 2008; Fallini et al., 2011). The expanding list of RBPs identified as SMN interactors together with the observation that SMN localizes in axons has put forward the hypothesis that SMN complexes distinct from those involved in snRNP biogenesis localize to axons to facilitate mRNA delivery followed by local protein synthesis to support axon growth and maintenance. Our study reveals that Smn interacts with the RBP Ptbp2 in axons. Ptbp2 primarily localizes to the cell body of neuronal cells but also is present in axons and growth cones, where it is involved in localization and translation of the *Hnrnpr* mRNA (Salehi et al., 2023). We observed that Smn regulates the axonal levels of Ptbp2 and the local synthesis of hnRNP R. This finding reveals an additional layer of complexity as the local production of RBPs such as hnRNP R might fine-tune local mRNA processing and translation. Loss of these regulatory pathways might destabilize axons, thereby contributing to the axonal pathology observed as an early pathological event in SMA. Conspicuously, we observed that Ptbp2 was reduced in proximal axons of Smn-deficient motoneurons while hnRNP R levels were more reduced in distal regions. While the exact mechanisms underlying this discrepancy are not known, it is possible that axonally localized Ptbp2 is not only derived from axonal transport, which is affected by Smn loss, but also from local synthesis of Ptbp2 in distal regions, which might not be affected by Smn deficiency.

How Smn modulates the axonal transport of Ptbp2 is currently not clear. It is possible that Smn associates with Ptbp2 already in the somata of motoneurons and that Smn-Ptbp2 complexes, together with the *Hnrnpr* mRNA, are transported in axons toward distal regions. In agreement with this notion, it has been shown previously that SMN interacts with the RBP HuD and that these proteins are actively co-transported in axons (Fallini et al., 2011). Alternatively, it is also conceivable that Ptbp2 bound to *Hnrnpr* mRNA associates with Smn locally in axons to facilitate hnRNP R synthesis. However, our PLA results show that Ptbp2 binds to Smn already in the somata of motoneurons, and it is thus more likely that Smn-Ptbp2 complexes are pre-assembled prior to transport. Either way, Smn-Ptbp2 complexes might be remodeled locally to induce translation of *Hnrnpr* mRNA, which might be kept in a translationally silent state during transport. In addition to *Hnrnpr* mRNA, the axonal localization of many other mRNAs might be regulated by Smn and Ptbp2. This possibility is supported by previous studies showing that there is a broad reduction of mRNAs in axons of Smn-deficient motoneurons (Fallini et al., 2011; Saal et al., 2014; Hennlein et al., 2023). Future experiments identifying the protein and RNA composition of axonal Smn and Ptbp2 complexes might reveal novel insights into the mechanisms whereby mRNAs are transported in axons to contribute to the proteomics diversity present at axon terminals.

In axons, Smn granules co-localize with ribosomal RNAs and control translation through interaction with other RBPs (Zhang et al., 2003; Lauria et al., 2020). An important finding of our study is that re-introducing Ptbp2 can rescue the axon growth defect of Smn-deficient motoneurons. We provide evidence that loss of Smn affects the local synthesis of hnRNP R and it is thus possible that deficiency of hnRNP R itself contributes to the axonal defects seen in motoneurons lacking Smn. Multiple lines of evidence indicate that hnRNP R is important for axon development. First, hnRNP R interacts with many mRNAs encoding proteins involved in axon growth and maturation including cytoskeletal components such as β-actin and synaptic proteins (Briese et al., 2018). Second, hnRNP R regulates the axonal localization of such transcripts to axons and it is possible that hnRNP R also controls their local translation (Briese et al., 2018). Third, depletion of hnRNP R reduces axon growth without affecting motoneuron survival (Glinka et al., 2010; Briese et al., 2018). Thus, by facilitating the local synthesis of hnRNP R, itself a multi-functional RBP, Smn together with Ptbp2 might stimulate axon growth. In addition to *Hnrnpr* mRNA, Ptbp2 has been shown to interact with many mRNAs (Licatalosi et al., 2012) suggesting that Smn-Ptbp2 complexes potentially regulate the axonal transport and local translation of several mRNAs.

We have previously shown that Ptbp2 promotes axonal translation of hnRNP R in cultured motoneurons by regulating the association of *Hnrnpr* mRNA with translating ribosomes (Salehi et al., 2023). Together with our finding that hnRNP R levels were reduced in axons but not cell bodies of Smn-deficient motoneurons, this suggests that Smn, through interaction with Ptbp2, promotes the axonal translation of hnRNP R in motoneurons for axon growth. Considering that neuromuscular connectivity is affected early in the course of SMA, our results thus highlight the importance of mechanisms for local protein synthesis for motoneuron development and functionality.

## Data Availability

The original contributions presented in this study are included in this article/Supplementary material, further inquiries can be directed to the corresponding author.

## Supplementary material

Supplementary Figure 1 Smn is associated with Ptbp2 in motoneurons. **(A,B)** Representative images of PLA signal in motoneurons at DIV 6 with either Ptbp2 antibody **(A)** or Smn antibody **(B)** alone as a negative control. Scale bars, 10 and 5 μm (magnified areas).
