# Supplementary figures and images for: Ptbp2 re-expression rescues axon growth defects in Smn-deficient motoneurons
Source: Front Mol Neurosci. 2024 Aug 23;17:1393779. doi: 10.3389/fnmol.2024.1393779 (PMC11377325; doi:10.3389/fnmol.2024.1393779)

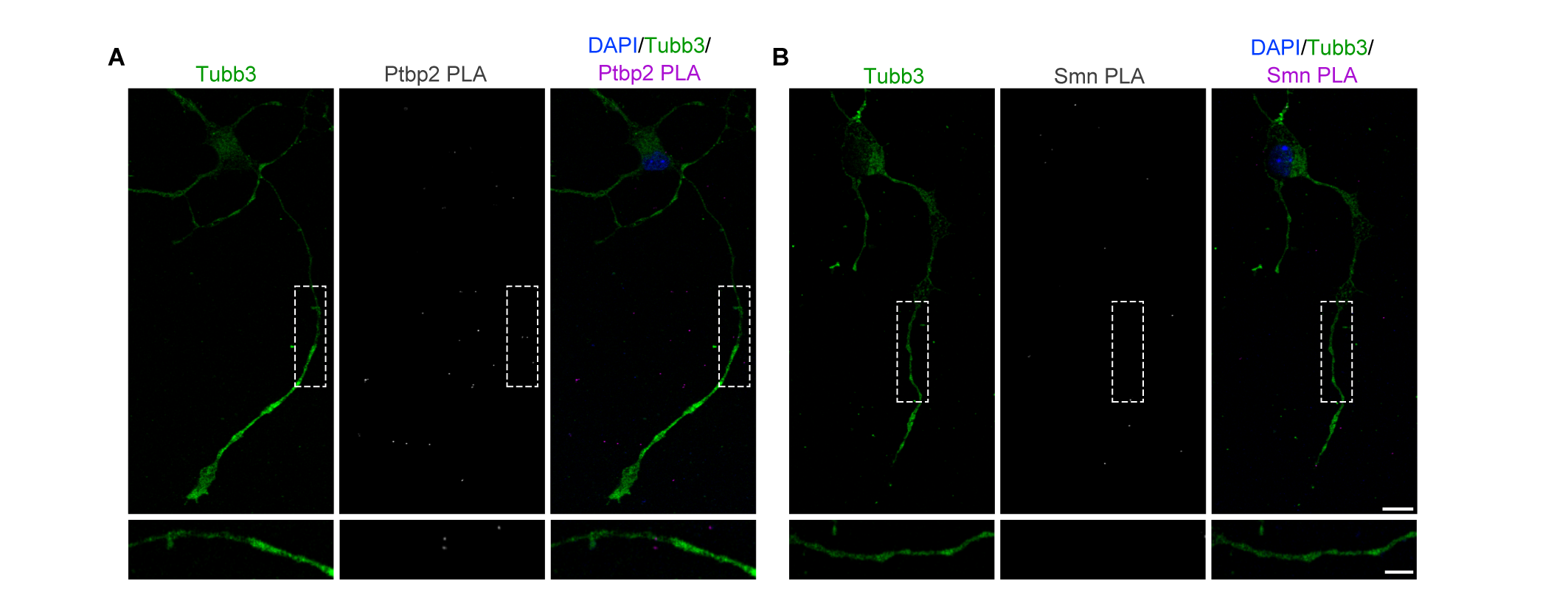

Supplement: Supplementary Figure 1 — Smn is associated with Ptbp2 in motoneurons. (A,B) Representative images of PLA signal in motoneurons at DIV 6 with either Ptbp2 antibody (A) or Smn antibody (B) alone as a negative control. Scale bars, 10 and 5 μm (magnified areas). [file Image_1.tif]
